# Impaired insulin sensitivity and reduced fertility in obese dairy cows

**DOI:** 10.1186/1751-0147-57-S1-O11

**Published:** 2015-09-25

**Authors:** Lisa Andrée O'Hara, Renée Båge, Kjell Holtenius

**Affiliations:** 1 Department of Animal Nutrition and Management, Swedish University of Agricultural Sciences, Uppsala, Sweden; 2 Department of Clinical Sciences, Swedish University of Agricultural Sciences, Uppsala, Sweden

## Introduction

The body condition score (BCS) at calving and the degree of BCS losses post-partum (PP) are associated with milk production, reproduction, and health. Collective data indicate that, at parturition, a BCS around 3.50 on a five graded scale, where 1 is emaciated and 5 is obese, will minimize BCS-related health and fertility disorders and allow high milk yield.

## Objective

The objective of this study was to evaluate the effects of body condition at parturition on the metabolism with focus on insulin sensitivity (IS) and on fertility as reflected by milk progesterone profiles.

## Material and methods

Multiparous cows (n=74) at the Swedish Livestock Research Centre Lövsta, with BCS from 2 to 4.25 the first week PP were enrolled. Insulin, non-esterified fatty acids (NEFA), glucose and insulin like growth factor 1 was determined week two PP. The Revised Quantitative Insulin Sensitivity Check Index (RQUICKI) was used to estimate IS. Progesterone in milk was determined twice weekly during weeks 2-12 PP.

## Results

IS was negatively related to BCS. None of the other measured plasma parameters were significantly affected. Among over-conditioned cows (BCS >3.75) 55 % showed progesterone profiles that deviated from normal. Corresponding figures for thin (BCS<3.25) and adequately (BCS 3.25-3.75) conditioned cows were 35 and 27% respectively.

## Conclusion

The negative relationship between BCS and IS supports studies indicating that overfeeding prior to parturition impaired IS. BCS was related to fertility, as reflected by progesterone profiles, in a non-linear manner indicating that fertility of both thin and over-conditioned cows was negatively affected.

